# Facile Fabrication of Graphene Oxide Nanoribbon-Based Nanocomposite Papers with Different Oxidation Degrees and Morphologies for Tunable Fire-Warning Response

**DOI:** 10.3390/nano12121963

**Published:** 2022-06-08

**Authors:** Wei-Wei Qiu, Zhi-Ran Yu, Ling-Yun Zhou, Ling-Yu Lv, Heng Chen, Long-Cheng Tang

**Affiliations:** 1 School of Information and Electronic Engineering, Zhejiang University of Science and Technology, Hangzhou 310023, China; zhiran.yu.etu@univ-lille.fr; 2 Key Laboratory of Organosilicon Chemistry and Material Technology of MoE, College of Material, Chemistry and Chemical Engineering, Hangzhou Normal University, Hangzhou 311121, China; 18758043323@163.com; 3 Guangdong Science & Technology Infrastructure Center, Guangzhou 510610, China; 4 College of Mechanical Engineering, Zhejiang University of Technology, Hangzhou 310014, China; hengchen@zjut.edu.cn

**Keywords:** graphene oxide nanoribbon, different oxidation degrees, layered structure, nanocomposite paper, fire-warning response

## Abstract

Smart fire-warning sensors based on graphene oxide (GO) nanomaterials, via monitoring their temperature-responsive resistance transition, have attracted considerable interest for several years. However, an important question remains as to whether or not different oxidation degrees of the GO network can produce different impacts on fire-warning responses. In this study, we synthesized three types of GO nanoribbons (GONRs) with different oxidation degrees and morphologies, and thus prepared flame retardant polyethylene glycol (PEG)/GONR/montmorillonite (MMT) nanocomposite papers via a facile, solvent free, and low-temperature evaporation-induced assembly approach. The results showed that the presence of the GONRs in the PEG/MMT promoted the formation of an interconnected nacre-like layered structure, and that appropriate oxidation of the GONRs provided better reinforcing efficiency and lower creep deformation. Furthermore, the different oxidation degrees of the GONRs produced a tunable flame-detection response, and an ideal fire-warning signal in pre-combustion (e.g., 3, 18, and 33 s at 300 °C for the three PEG/GONR/MMT nanocomposite papers), superior to the previous GONR-based fire-warning materials. Clearly, this work provides a novel strategy for the design and development of smart fire-warning sensors.

## 1. Introduction

Fire is a “double-edged sword” since it has been of great significance in the history of human civilization, but is also hazardous [1,2], especially since the development of synthesized polymer. As is well known, London’s Grenfell Tower fire in 2017, Brazil’s National Museum fire in 2018, and Paris’ Notre Dame fire in 2019 have caused massive casualties, irreparable property damage, and the loss of priceless artefacts [2,3,4]. These severe outdoor fire disasters have been attributed to the high fire-risk of various combustible materials that have low ignition temperatures of 300–500 °C and rapid flame-spread speeds (e.g., about 8 m for <80 s in the large-scale UL experiment [5]). Some traditional fire alarm strategies, including smoke alarms and heat detectors, have proven to be effective at significantly reducing or even avoiding the risk of indoor fires; however, they have limitations, including a relatively long fire alarm time of >100 s, no fire early-warning signal below ignition temperature, and restricted use in certain outdoor environments [6]. Therefore, understanding how to monitor the critical outdoor fire risk of combustible materials is imperative, but remains a challenge.

To address the above issues, novel and complementary fire-warning materials and sensors have been developed over several years. Typically, the temperature-responsive resistance transition of various nano-fillers, e.g., graphene oxide (GO) [7,8,9,10,11,12,13,14], carbon nanotube (CNT) [15], and MXene [16], has been widely used to construct sensitive fire-warning sensors. Among them, GO-based fire-warning materials with sensitive flame detection and fire early-warning response have attractive considerable research interest. Typically, pristine GO networks are electrically insulated due to oxygen-containing functional groups in their structure [17,18,19]. On encountering a flame, the reduction of oxygen groups in the insulating GO network can chang it into an electrically conductive rGO one [20,21,22], thus providing a rapid flame-detection response time (normally less than 10 s [11,14,23,24,25,26,27]). On the other hand, GO-based fire-warning materials also offer an ideal fire early-warning signal that can be activated below the ignition temperatures of most flammable materials, e.g., 232 s at 200 °C and 35 s at 300 °C for _3_-mercaptopropyltrimethoxysilane modified-GO nanocomposite paper [13]. A sensitive fire early-warning response is strongly dependent on the formation of the interconnected network and the oxidation degrees of the prepared GO sheets. Moreover, the response of such sensors should also depend on the environmental atmosphere, and this will be further investigated.

Among GO derivatives, GO nanoribbon (GONR) shows promise for achieving rapid and sensitive fire-warning response signals during the precombustion process, owing to the effective formation of an interconnected network. Our previous work demonstrated that GONRs produced more rapid resistance-response behaviors than the corresponding GO sheets and GO wide ribbons (GOWR) [28,29]. At an unchanged temperature of ∼300 °C, these fire-warning materials and sensors showed different early-warning response times (to activate the alarm light) of 24.0, 33.5, and 39.0 s for the GO nanoribbon, the GO wide ribbon, and the GO sheet at the same concentration, respectively. This was attributable to the formation of a highly interconnected GONR network, and to GONR thermal reduction behavior that was more effective than that of the other two GO derivatives (i.e., GOWR and GO sheet). Thus, based on the thermal reduction feature of GONR networks [30], different oxidation degrees are crucial to determining the final fire-warning performance of the fire-warning sensor, and it is therefore necessary to understand the effect of oxidation degrees on the two important parameters (i.e., flame detection response time and fire early-warning response time). Whether or not the different oxidation degrees of GONRs can result in different impacts on temperature-induced resistance transition behaviors remains an important question.

In the present work, we synthesized three types of GONR with different oxidation degrees and morphologies, and thus prepared GONR-based nanocomposite papers via a facile and low-temperature evaporation-induced assembly approach. The oxidation degrees and morphologies of the GONRs were analyzed and discussed by various characterizations. The appropriate oxidation degree of the GONRs, along with polyethylene glycol (PEG) molecules, produced a better-oriented montmorillonite (MMT) structure than the other two GONRS, which produced different impacts on the mechanical properties and fire-warning performance. The structure, mechanical properties, and fire-warning response of the three GONR-based nanocomposite papers were investigated and compared.

## 2. Materials and Methods

### 2.1. Materials

Montmorillonite (MMT) with purity >98%, and multi-walled carbon nanotubes (MWCNTs) with 20–50 nm in outer diameters, were purchased from Nanocor Co., Ltd., Aberdeen, MS, USA, and Najing Xianfeng Nanomaterials Technology Co., Ltd., Nanjing, China, respectively. Other reagents including potassium permanganate (KMnO_4_), PEG with molecular weight of 6000, concentrated sulfuric acid (H_2_SO_4_, ≥98 wt%), and hydrogen peroxide (H_2_O_2_, 30 vol%); filter paper was supplied by Sinopharm Chemical Reagent Co., Ltd., Hangzhou, China.

### 2.2. Preparation of GONR Fillers with Different Oxidization Conditions

GONR aqueous solution was prepared by longitudinal unzipping of MWCNTs;the detail of the process can be found in Refs. [30,31,32]. Notably, the GONRs with three oxidation degrees (named GONR-1, GONR-2, and GONR-3) were easily prepared by adjusting the oxidation time and the concentration of the KMnO_4_ during the unzipping process. Typically, 5.0 g MWCNTs were added into 2000 mL of concentrated H_2_SO_4_, and sonicated for 0.5 h, then stirred for 1.0 h at room temperature. After that, 12.5 g KMnO_4_ was added to the above dispersion, stirred for 1.0 h, and sonicated for 0.5 h at room temperature. Then, the above mixture was stirred for 1.0 h at room temperature, and then heated to 70 °C for another 1.0 h, with continuous stirring, and quenched into a mass of ice-cold distilled water with excessive hydrogen peroxide (H_2_O_2_, 30%). The reaction was kept overnight. After that, the dispersion was centrifuged and washed with 5% aqueous hydrochloric acid (HCl), to remove metal ions several times. Finally, the mixture was washed with distilled water, to remove acid, and dialyzed in deionized water for several days to reach a neutral or weak acidic state. As a result, the GONR-1 filler was obtained. For adjusting the oxidation conditions, the weight of KMnO_4_ was changed into 25.0 g by the same procedure as described above, and the GONR-2 was then prepared. To further oxidate the CNT, the oxidation time after adding 25.0 g of KMnO_4_ was extended to 105 min, and the prepared GONR was then named as GONR-3.

### 2.3. Preparation of GONR-Based Nanocomposite Papers

The GONR nanocomposite paper was fabricated by using the following process. Firstly, the PEG molecules were dissolved into water, to obtain a uniform dispersion, and the PEG concertation was kept at ~2.5 mg/mL. Then, the highly dispersed clay/water solution was ultrasonically dispersed in the above PEG solution until a stable PEG/MMT suspension was obtained. After that, the GONR aqueous solution (5 mg/mL) and 5.0 wt% tannic acid/water solution were slowly added into the mixture, by stirring, to get a highly dispersed PEG/MMT/GONR/water solution. The above solution was then put into a petri dish with a diameter of 80 mm, and dried at 50 °C for 24 h, which made the MMT and GONR assemble into the free-standing paper. For comparison, the PEG/MMT/CNT paper was also prepared by the same fabricating process as described above. According to our previous work, the optimized ratio of GONR/MMT/PEG was about 1/1/1. The designation of the nanocomposite papers with different oxidation degrees of GONR was symbolized as G_x_, with x standing for the type of the GONR. For example, G_1_ indicated the paper containing 1 phr GONR-1,1 phr PEG, and 1 phr MMT.

### 2.4. Characterizations

The structure and morphology of the CNT and GONR fillers, and GONR-based nanocomposite papers, were examined by scanning electron microscopy (Zeiss Ultra Plus and HITACHI S-4800). Fourier transform infrared spectra of various fillers were conducted by using a Nicolet 7000 FTIR (Nicolet Instrument Company, Waltham, MA, USA) between 500 and 4000 cm^−1^. Thermogravimetric (TG) analysis was made by using a TA Instruments Q500, and the temperature was conducted from room temperature to 800 °C in air atmosphere (at a heating rate of 10 °C/min). Raman spectra were recorded with SENTERRA Micro Raman Spectroscopy (Bruker Instrument, Rheinstetten, Germany). The excitation wavelength was 633 nm from a He-Ne laser with a laser power of ca. 15 mW at the sample surface. X-ray diffraction (XRD) was performed on various fillers and nanocomposites. The measurements were conducted at a scan rate of 5 min^−1^ between 2 and 4° using a D/Max 2550V X-ray diffractometer (Rigaku, Tokyo, Japan).

The mechanical properties and creep-recovery performance of various nanocomposite papers were measured by DMA Q800, and the maximum load cell was 18 N, with loading speed of 1 mm/min. All the samples were cut into 20 mm in length and 3 mm in width, and the sample thickness values were measured by using SEM imaging. Five specimens for each sample were conducted, to obtain the average value of the mechanical properties. Creep and recovery tests of the three GONR-based nanocomposite papers were performed by using a dynamic mechanical analyzer (TA Instruments Q800, New Castle, DE, USA). The specimens for creep tests were the same size as for the DMTA tests. At least three samples were tested for each composition. The creep strain was determined as a function of the time (creep = 600 s and recovery = 600 s) under both R.T. and 80 °C. The applied 10 MPa stress corresponded to approximately 30% of the tensile strength of the samples [33], so as to ensure creep measurements in the linear viscoelastic deformation regime. For the fire alarm sensor, the optimized nanocomposite papers were directly connected with a DC electric source of 12 V and an alarm light. Silver paste was used to connect the copper electrodes and the alarm light, so as to reduce the contact resistance. The electrical resistance values of the papers in the high-temperature environment were tested by a picoammeter (6487, Keithley Instruments, Cleveland, OH, USA).

## 3. Results

### 3.1. Dispersion and Structure of GONR Fillers with Different Oxidation Degrees

**Figure 1:**
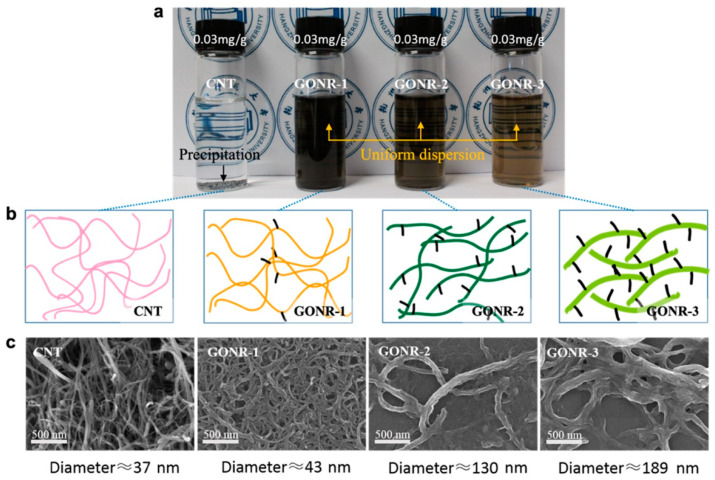
Dispersion and morphology of the CNT and GONRs with different oxidation degrees: (**a**) photos of dispersion of the CNT and GONRs in aqueous solution, showing different dispersion levels; (**b**) schematic illustration of the CNT and GONRs with different oxidation degrees; (**c**) SEM images of the CNT and GONRs with different morphology and structure features.

Figure 1 shows the dispersion and structure of the raw CNT and GONRs with different oxidation degrees in aqueous solution. As shown in Figure 1a, compared with the obvious precipitation of the CNT in the water, the GONRs showed uniform dispersion in the aqueous solution, although they presented different colors. Normally, pure CNT has few oxygen groups, which would lead to poor dispersion stability in aqueous solution. Comparatively, by increasing the content of KMnO_4_ oxidant and the oxidizing time, more and more oxygen groups attached to the CNT, and produced better dispersion and stability (as illustrated in Figure 1b). The different oxidation degrees of the GONRs will be discussed in the following section. Furthermore, the SEM images of the CNT and GONRs also demonstrated different morphologies and structure features. As shown in Figure 1c, compared with the 1D MWCNT nanotubes with 20–50 nm, the GONR-1 tubes exhibited a certain interfacial interaction, since they were tangled together, and the average diameter of the GONR-1 showed a slight increase from 37 nm for pure CNT to ~43 nm. Notably, if a higher oxidation degree was applied, a better unzipping degree of the CNT was obtained, thus producing a much larger diameter of the prepared GONR. Typically, the diameter values of GONR-2 and GONR-3 reached ~130 and 189 nm, which was about 3–5 times greater than that of the pure CNT, further indicating the effective unzipping effect of the CNT. Moreover, some nano-scale rough structures were visible on these GONR-2 and GONR-3 fillers, which was likely due to the existence of many oxygen-containing functional groups (e.g., carboxyl and hydroxyl groups) on the filler surface [34,35].

**Figure 2:**
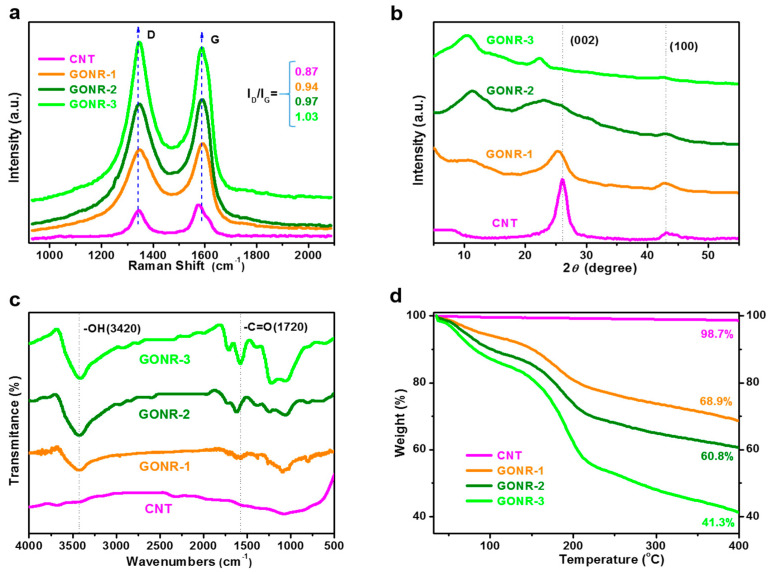
Structural characterization and analysis: (**a**) Raman spectra; (**b**) XRD curves; (**c**) FTIR spectra; (**d**) TGA curves of CNT and GONRs with different morphology and structure, indicating the different oxidation degree.

To demonstrate the different oxidation degrees of the GONRs prepared in this manuscript, Raman spectra and XRD curves were conducted to understand their differences. Figure 2a shows the Raman spectra of the CNT and GONRs with different oxidation degrees. The Raman spectrum of the pure CNT displayed a prominent G-peak as the feature at 1581 cm^−1^, and a D-band adsorption at 1335 cm^−1^, which were ascribed to the first-order scattering of the E_2g_ vibration mode and to the defects inherent in the CNT structure and the edge effect of the CNT (A_1g_ mode) [36]. There was obvious structural change during the oxidation from the CNT to the GONRs: the D-band acquired higher relative intensity, showing an increased D/G intensity ratio from 0.87 for pure CNT to 0.94, 097, and 1.03 for GONR-1, GONR-2, and GONR-3, respectively. This indicated that distortion of the bonds and the destruction of symmetry, possibly due to the reduction in size of the in-plane sp^2^ domains caused by the effective oxidation; the higher oxidation degree reflected the smaller average size of the sp^2^ domains upon oxidation of the CNT [37]. The XRD curves of the above fillers further demonstrated this. With the increase of unzipping and oxidation degree, the 002 peak of the CNT gradually shifted to low values (Figure 2b), due to more oxygen groups attaching to the CNT, and thus increasing the layer spacing [38].

To further illustrate the above observed oxidation degrees of the GONRs prepared in this work, FTIR and TGA results of the CNT and three types of GONRs were characterized. Figure 2c shows the FTIR spectra of the CNT and GONRs. Compared to the pure CNT with no characteristic peaks, oxygen-containing functional groups gradually appeared, e.g., O-H at 3420 cm^−1^ and C=O at 1720 cm^−1^, and the peak intensity showed an obvious increase with the increasing of the oxidation degree. This was consistent with the above Raman spectra and XRD results. TGA curves of various fillers were also conducted, and the results are shown in Figure 2d. As expected, the pristine CNT exhibited high thermal stability, and did not decompose up to 400 °C [32]. After oxidation, the GONRs showed significant decomposition at approximately 160–300 °C under air atmosphere, which was likely due to the pyrolysis of the unstable oxygen functionalities (such as the hydroxyl, carbonyl, and carboxylic groups) generating gases including CO, CO_2_, and steam [39,40]. In particular, the residual weight values at 400 °C were different, e.g., 68.9 wt%, 60.8 wt%, and 41.3 wt% for GONR-1, GONR-2, and GONR-3, respectively. The above results confirmed that the prepared GONR-1, GONR-2, and GONR-3 had different oxidation degrees.

### 3.2. Preparation and Structure of GONR-Based Nanocomposite Papers

**Figure 3:**
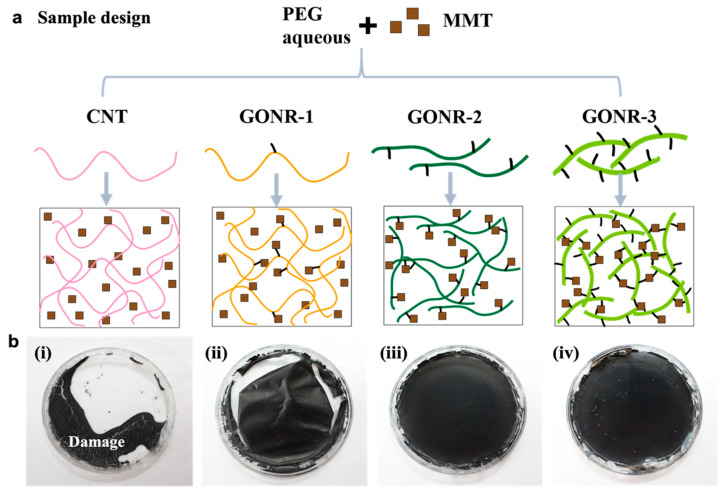
Design and fabrication of PEG/GONR/MMT nanocomposite papers: (**a**) design of the PEG/GONR/MMT nanocomposite papers with different GONR derivatives; (**b**) photos of the PEG/GONR/MMT nanocomposite papers: (**i**) CNT; (**ii**) GONR-1; (**iii**) GONR-2; and (**iv**) GONR-3.

To fabricate the flame-retardant GONR-based papers, water-soluble PEG molecules and layered MMT were used via a water-based and solvent-free solution, followed by the low-temperature evaporation-induced assembly (EISA) approach. Figure 3 shows the schematic illustration of the fabrication and morphology of the GONR-based nanocomposite papers with the CNT and three types of GONRs. Normally, the MMT and PEG molecules form a free-standing paper after the EISA process under 50 °C for 24 h. As shown in Figure 3b(i), however, the presence of pure CNT caused the structure shrinkage to destroy the structure of the paper, which was likely due to the poor dispersion of CNT in the PEG matrix. Owing to the many hydroxyl groups on the GONR/MMT sheets and the PEG molecules, the formation of weak hydrogen bonding and van der Waals interactions among them induced an effective interconnected network in the hybrid organic/inorganic artificial nacre-like nanocomposite papers [41]. Furthermore, with the increase of the oxidation degree from GONR-1 to GONR-2 and GONR-3, the nanocomposite papers showed more and more structural integrity (ii, iii, and iv in Figure 3b), which was attributable to the formation of interactions between the oxygen groups of GONRs and PEG or MMT fillers, and to the effectively interconnected structure in the layered MMT fillers [30].

**Figure 4:**
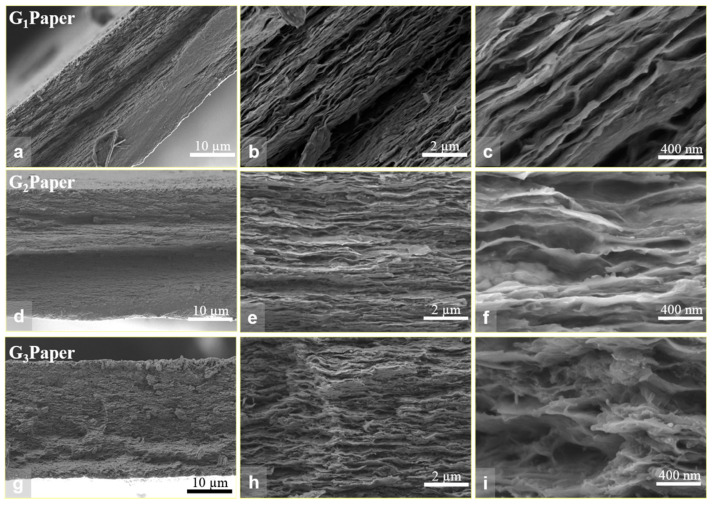
Morphology and structure of PEG/GONR/MMT nanocomposite papers: (**a**–**c**) PEG/GONR-1/MMT (G_1_) paper; (**d**–**f**) PEG/GONR-2/MMT (G_2_) paper; (**g**–**i**) PEG/GONR-3/MMT (G_3_) paper, showing nacre-like layered structure.

To explore the morphology and microstructure of the PEG/GONR/MMT networks, SEM fracture surface images of the G_1_, G_2,_ and G_3_ nanocomposite papers were performed. Figure 4 shows the typical SEM images of the PEG/GONR/MMT nanocomposite papers. Typically, the ternary G_1_ nanocomposite paper displayed well-oriented MMT structure (Figure 4a,b), although some obvious gaps were visible on the fracture surface, probably due to the debonding sheets during the tensile process (Figure 4c). Comparatively, the fracture morphologies of the G_2_ and G_3_ nanocomposite papers showed certain differences. Firstly, although well-oriented MMT structures were present on the fracture surface, the gaps between the MMT sheets were obviously smaller (Figure 4d,e,g,h), implying the formation of effective interfacial interactions among the fillers and the PEG matrix. Secondly, careful observation suggested that some PEG molecules and GONRs were well-embedded in the G_2_ and G_3_ nanocomposite papers (Figure 4f,i), and that the oriented degree of MMT and compact structure in the G_2_ paper seemed to be better than the other two papers, although the GONRs could hardly be seen, probably due to good compatibility between the hydroxyl groups of GONRs and MMT [30].

### 3.3. Mechanical Property

**Figure 5:**
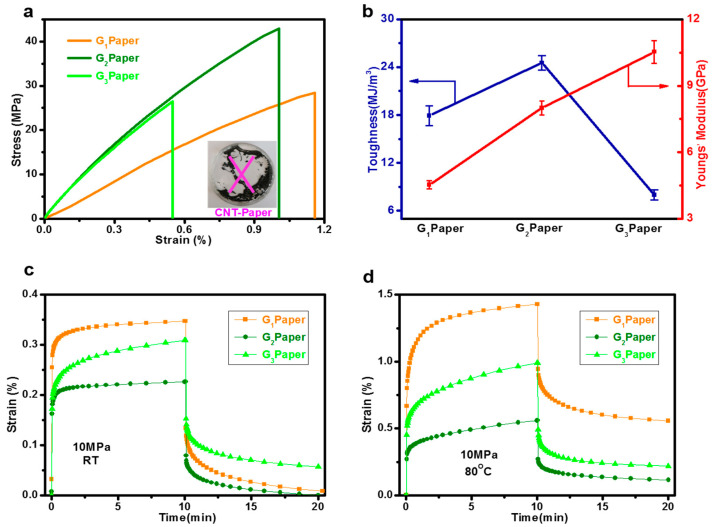
Mechanical properties and creep-recovery of PEG/GONR/MMT nanocomposite papers: (**a**) typical tensile stress-strain curves; (**b**) Young’s modulus and toughness; (**c**,**d**) creep and recovery curves of PGM papers under R.T. and 80 °C.

Typically, the pristine CNT in the PEG and MMT did not form a compact network, and the prepared CNT-based paper was completely damaged (see the inset in Figure 5a), while the presence of GONRs with different oxidation degrees in the PEG and MMT network produced free-standing nanocomposite papers (Figure 3b). The typical stress-strain curves of the G_1_, G_2,_ and G_3_ nanocomposite papers are shown in Figure 5a, and the results of Young’s modulus and toughness are listed in Figure 5b. Clearly, the GONRs with different oxidation degrees produced different mechanical properties. The ultimate tensile strength and elongation at break of the G_1_ nanocomposite paper were about 27 MPa and 1.16%, respectively. Comparatively, the G_2_ nanocomposite paper showed enhanced tensile strength (~42 MPa) and slight decrease in elongation at break (0.95%). Notably, the addition of GONR-3 induced simultaneous reduction in both the tensile strength (~26 MPa) and elongation at break (0.55%), although Young’s modulus displayed much higher than the other two nanocomposite papers. The toughness values of the papers, calculated by integrating the stress-strain curves in Figure 5b, indicated that the addition of GONR-2 produced the highest toughness value among the GONR-based nanocomposite papers.

To further explore the effect of the GONRs with different oxidation degrees on the mechanical performance of the nanocomposite papers, their creep-recovery behaviors were measured and compared. As shown in Figure 5c, the creep strain values increased after applying a stress value of 10 MPa for 10 min at room temperature. Among the three samples, the G_2_ paper presented the lowest creep strain and slowest creep rate when compared with those of the G_1_ and G_3_ papers. These results were supported by the well-oriented MMT structure in Figure 4, that could endure low stress value effectively. Moreover, the creep strain of the G_2_ paper completely recovered after removing the applied stress for 10 min, confirming the outstanding mechanical elasticity. Similar creep strain and recovery behaviors of the three nanocomposite papers were also observed under a high temperature of 80 °C. As shown in Figure 5d, although the high temperature was able to accelerate the creep strain from 0.20 to 0.35% at R.T. to 0.40–1.45%, the change in creep strain values of the three nanocomposite papers was almost same. The G_2_ paper displayed about 0.50% creep strain after applying 10 MPa under 80 °C for 10 min, while the G_1_ and G_3_ papers showed 1.00% and 1.45% creep strain values, respectively. The recovered deformation of the G_2_ paper after 10 min was the lowest value among the three samples investigated, indicating an effective recovery feature.

### 3.4. Fire-Warning Response

**Figure 6:**
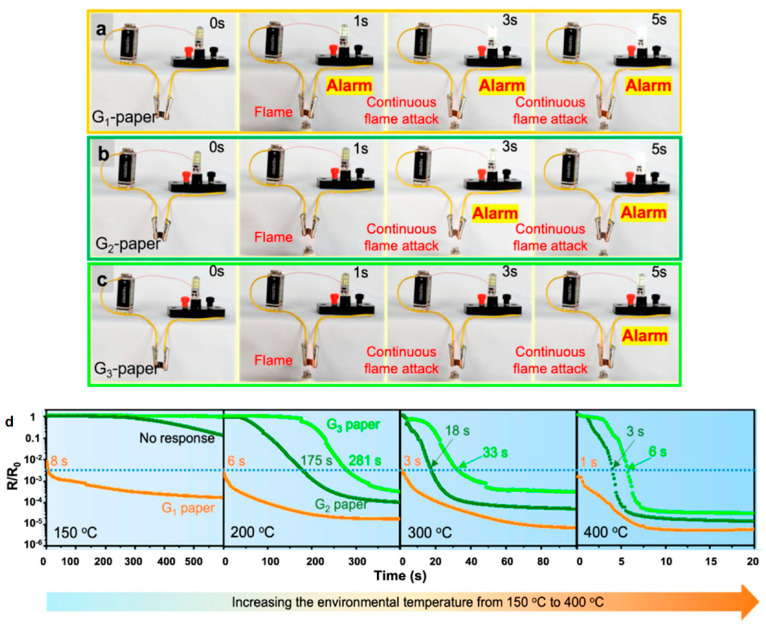
Fire-warning response behavior of PEG/GONR/MMT nanocomposite papers: (**a**–**c**) flame-detection response of G_1_, G_2_, and G_3_ nanocomposite papers; (**d**) resistance change of the nanocomposite papers under different temperature values, from 150 °C to 400 °C.

The formation of a GONR network in the PEG/MMT would provide an ideal fire-warning response, owing to the resistance transition from an insulating state to a conductive one, when encountering a flame source or abnormally high temperature environment, as a GO-based fire-warning sensor [7,13,14,29,42,43,44,45]. The fire-warning sensor was prepared by connecting the nanocomposite papers with an alarm light and a low-voltage electric source with ~12 V via a wire, as shown in Figure 6a. All the prepared nanocomposite papers exhibited excellent good structural stability after exposure to the flame, and no structural damage was observed in Figure 6a–c, confirming excellent flame resistance [46]. The above phenomena ensured a stable fire-warning release during the flame-detection process. Notably, the different oxidation degrees of the GONRs were able to produce rapid and tunable flame-detection response time. For the G_1_ paper, on exposing the paper to the flame, the alarm light was triggered in only 1 s, and a continuous fire alarm signal was obtained. Comparatively, although the G_2_ and G_3_ papers presented similar structural integrity during the flame attack, the flame-detection response time exhibited slightly delayed phenomena, e.g., 3 s for G_2_ and 5 s for G_3_, respectively. During the flame-detection process, the interconnected GONR network in the nanocomposite papers was rapidly thermally reduced into conductive rGONR, thus triggering the alarm light, which offered reliable and timely fire alarm signals [29]. The different oxidation degrees of the GONRs shown in Figure 2d tuned the response time of the sensor effectively.

To further demonstrate the resistance change, the electrical conductivity values of the three nanocomposite papers as a function of time were measured under a different temperature range of 150–400 °C; the results are shown in Figure 6d. Clearly, the electrical resistance of the GONR network in the nanocomposite paper was also dependent on the applied temperature. Under 150 °C, the resistance of the G_1_ paper decreased significantly in a very short time, which triggered the alarm light rapidly (2.5 orders of magnitude in 7–10 s). Such low temperature is below the ignition temperatures (300–500 °C) of most flammable materials, indicating that the ideal fire-warning signal can be obtained via monitoring the resistance change of the G_1_ nanocomposite paper. However, the G_2_ and G_3_ papers displayed only slight changes in resistance, suggesting that the higher oxidation of the insulating GONRs was barely thermally reduced to conductive rGONR under 150 °C. By increasing the temperature from 150 to 400 °C, the transition phenomena in the electrical resistance of the three nanocomposite papers was much more obvious as a function of time. The response time to activate the light (resistance change of 2.5 orders of magnitude, the dotted line) was strongly dependent on the oxidation degrees of the GONRs. As shown in Figure 6d, the response time values of the G_1_, G_2_, and G_3_ papers were about 6, 175, and 281 s under 200 °C, respectively; and they were reduced to 3, 18, and 33 s under 300 °C and 1, 3, and 6 s under 400 °C, respectively. The rapid and sensitive fire-warning response of the G_1_ and G_2_ papers was superior even to the previous GONR-based fire-warning materials [30]. These results confirm that the lower oxidation degree of GONRs can release a much quicker fire-warning response time. Therefore, this work offers a promising approach to effectively adjusting the fire-warning response time of nanocomposite papers via simply altering the oxidation degree of the GONRs, so as to provide a timely and active fire-warning response before a fire accident.

**Figure 7:**
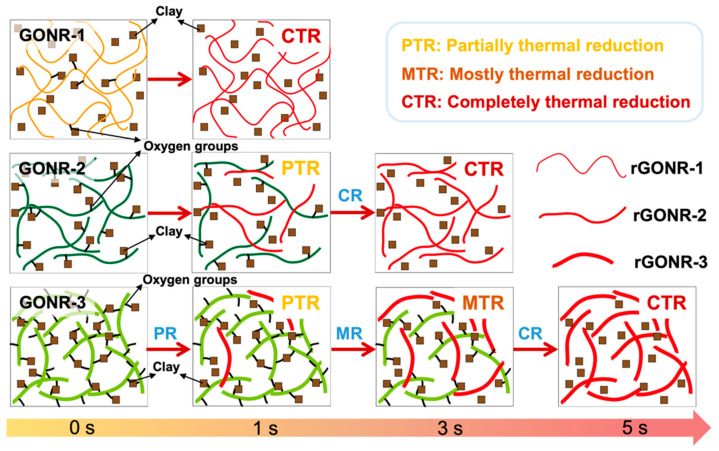
Schematic illustration of fire-warning mechanisms of nanocomposite papers with different oxidation degrees of GONRs under high-temperature conditions.

To clearly explain the resistance transition mechanisms of PEG/MMT/GONR, a schematic illustration is proposed and shown in Figure 7. Typically, in such flame source or high-temperature environments, the GONR network in the nanocomposite papers can be quickly thermally reduced into conductive rGONR, thus activating a current flow to go through the alarm light, which offers reliable and timely fire alarm signals. The different oxidation degrees of the GONR network should produce different thermal reduction behaviors under high temperature conditions. As proposed in Figure 7, the insulating GONR-1 with few oxygen groups would be completely and quickly reduced, to generate an effective resistance transition and thus form a conductive rGONR network. Comparatively, the higher oxidation degree of the GONR would delay the reduction rate of the GONR network, and thus induce a partial and mostly thermal reduction process, especially at high temperature conditions. This is well-supported by the TGA results in Figure 2d. Of course, further research must be conducted, to clarify the thermal reduction behaviors of the different oxidized GONR, which will be addressed by our future work.

## 4. Conclusions

In summary, we prepared the PEG/MMT/GONR nanocomposite papers with different oxidation degrees and morphologies via a facile, solvent free, and low-temperature evaporation-induced assembly approach. Three different oxidation degrees and morphologies of GONRs were synthesized via adjusting the concertation of the starting oxidant and the oxidation time. The combined use of GONR and MMT, along with PEG molecules, formed an interconnected nacre-like layered structure, and the GONR-2 produced a better-oriented MMT structure. As a result, the mechanical properties showed that the appropriate oxidation of GONR-2 produced an effective reinforcing efficiency. Typically, in comparison with the G_1_ and G_3_ papers with tensile strength of about 27 MPa and 26 MPa, the tensile strength of the G_2_ nanocomposite paper was about 42 MPa. Such artificial nacre of the EG/MMT/GONR nanocomposite paper exhibited an excellent flame-retardant property and showed tunable flame-detection response and sensitive fire early-warning response before being ignited (e.g., 6, 175, and 281 s at 200 °C for the G_1_, G_2_ and G_3_ papers). Considering the balance between the mechanical property and fire-warning response, the appropriate oxidation degree of GONR is promising for fabricating a fire-warning sensor for potential application. The response time of the fire-warning sensor can be effectively tuned by using the different oxidation degrees of GONRs for monitoring critical fire risk, which would provide a new way to construct a sensitive fire-warning sensor.

## Data Availability

The data presented in this study are available on request from the corresponding author.
